# Development of a Ferritin Protein Nanoparticle Vaccine with PRRSV GP5 Protein

**DOI:** 10.3390/v16060991

**Published:** 2024-06-20

**Authors:** Xinjian Chang, Jun Ma, Yanrong Zhou, Shaobo Xiao, Xun Xiao, Liurong Fang

**Affiliations:** 1 National Key Laboratory of Agricultural Microbiology, College of Veterinary Medicine, Huazhong Agricultural University, Wuhan 430070, China; changxj@webmail.hzau.edu.cn (X.C.); ma.jun@webmail.hzau.edu.cn (J.M.); yrzhou@mail.hzau.edu.cn (Y.Z.); vet@mail.hzau.edu.cn (S.X.); 2 The Key Laboratory of Preventive Veterinary Medicine in Hubei Province, Cooperative Innovation Center for Sustainable Pig Production, Wuhan 430070, China

**Keywords:** PRRSV, ferritin, subunit vaccine, virus-like particle

## Abstract

Porcine reproductive and respiratory syndrome virus (PRRSV) presents a significant threat to the global swine industry. The development of highly effective subunit nanovaccines is a promising strategy for preventing PRRSV variant infections. In this study, two different types of ferritin (Ft) nanovaccines targeting the major glycoprotein GP5, named GP5m-Ft and (Bp-IVp)_3_-Ft, were constructed and evaluated as vaccine candidates for PRRSV. Transmission electron microscopy (TEM) and dynamic light scattering (DLS) demonstrated that both purified GP5m-Ft and (Bp-IVp)_3_-Ft proteins could self-assemble into nanospheres. A comparison of the immunogenicity of GP5m-Ft and (Bp-IVp)_3_-Ft with an inactivated PRRSV vaccine in BALB/c mice revealed that mice immunized with GP5m-Ft exhibited the highest ELISA antibody levels, neutralizing antibody titers, the lymphocyte proliferation index, and IFN-γ levels. Furthermore, vaccination with the GP5m-Ft nanoparticle effectively protected piglets against a highly pathogenic PRRSV challenge. These findings suggest that GP5m-Ft is a promising vaccine candidate for controlling PRRS.

## 1. Introduction

Porcine reproductive and respiratory syndrome (PRRS) is a highly contagious disease that significantly threaten to the health of pig herds [1,2,3]. Generally, PRRSV consists of two basic species: Betaarterivirus suid 1 (PRRSV-1 or the European genotype), and Betaarterivirus suid 2 (PRRSV-2 or the North American genotype) [4,5]. The North American genotype has caused a worldwide pandemic, resulting in severe economic damage to the pig industry [6,7]. Vaccination plays a crucial role in controlling the spread of PRRSV. To date, various types of PRRSV vaccines have been approved and utilized, including modified live vaccines and inactivated vaccines [8,9,10]. The inactivated vaccines are known for their safety, lack of risk, and ease of storage and transportation. However, multiple vaccinations are required, and they may only provide limited immune protection. On the other hand, modified live vaccines can elicit a strong immune response with a long-lasting immunity period. Nevertheless, there is a potential risk of regaining virulence and potential recombination with virulent field strains, as well as an inability to fully protect pigs from heterogenous strains. While existing vaccines have shown some effectiveness, there remains a need to develop a safer and more efficient vaccine. Genetically engineered subunit vaccines have garnered significant attention due to their safety profile and ease of production [11,12].

The primary structural genes of PRRSV consist of ORFs 2–7, which encode eight structural proteins, including the minor envelope proteins (GP2a, GP3, GP4, E, and ORF5a), major envelope proteins (GP5 and M), and the nucleocapsid protein (N) [13,14]. The GP5 protein of PRRSV is a multi-transmembrane protein, with its N- and C-terminal extracellular regions playing a critical role in the generation of neutralizing antibodies. Several research groups have identified two well-known neutralizing epitopes (GP5B and GP5IV) within these regions [15,16]. Numerous studies have demonstrated that the neutralizing antibodies induced by these two epitopes can effectively reduce the lethality of PRRSV infection in pigs and are, therefore, important targets for the development of subunit vaccines [17,18,19]. Although GP5 is considered to be an ideal target for the design of novel vaccines, some experimental vaccines based on the expression of the native GP5 protein, such as subunit vaccines, viral vector vaccines, DNA vaccines, and so on, often fail to induce higher neutralizing antibodies [18,20,21,22,23,24,25]. Additionally, there is also a non-neutralizing decoy epitope (aa 27–31) located upstream of the neutralizing epitope GP5B (aa 37–45), which may hinder the recognition of the GP5B epitope and the subsequent development of neutralizing antibodies [15]. Therefore, we replaced the decoy epitope with the GP5B epitope and named the mutant gene GP5m. At the same time, we designed another antigen gene, Bp-IVp, by linking together the GP5B (aa 37–45) epitope, Plasmodium generalist T-cell epitope (PADRE) [26], and the GP5IV epitope (aa 186–200) [16].

Ferritin is a spherical iron storage protein that is widely distributed in organisms and consists of 24 subunits. These subunits can self-assemble into a spherical hollow nanocage with an outer diameter of 12 nm and an inner cavity diameter of 8 nm [27,28,29]. In recent years, ferritin has been extensively employed as a nanocarrier in the development of various viral vaccines, including those for influenza virus, classical swine fever virus, human immunodeficiency virus, porcine rotavirus, and others [30,31,32,33,34]. However, there are few reports on the use of ferritin as a vector to develop PRRSV vaccines. Ma [21] successfully fused modified GP5 with ferritin using the baculovirus system, and subsequently expressed and prepared the GP5m-Ft nanoparticle vaccine. Their work demonstrated that the GP5m-Ft subunit vaccine was capable of inducing specific protective immune responses, indicating its potential as a promising vaccine candidate. However, our research has taken a different approach based on the work of Ma et al. We opted for *E. coli* as the expression system and achieved high-level expression of the GP5m-Ft protein. Additionally, we utilized aluminum hydroxide as an adjuvant to assess its immunological effect. Notably, we employed a dual model involving mice and piglets to comprehensively verify the immunogenicity of the protein. This strategy allowed for a more accurate evaluation of vaccine performance in diverse animal models, providing a more reliable foundation for clinical vaccine applications. Furthermore, we designed a novel antigen by cascading the B and IV epitopes of the GP5 protein (aa 186–200) and inserting them into a Plasmodium universal T cell epitope (PADRE). This resulted in an antigen with enhanced immunogenicity. 

At present, the existing PRRS vaccines are limited in their ability to address the high mutation rate and immune evasion of the virus. They exhibit low immunogenicity, provide minimal cross-protection, and may pose potential safety concerns. Consequently, effectively addressing the complex challenges posed by the high virus mutation rate and immune evasion is difficult. Therefore, this study aims to develop an innovative HP-PRRSV homologous vaccine with strong immunogenicity and high safety. This will serve as a foundation for further development of PRRS vaccines.

In this study, we designed two GP5-associated ferritin subunit vaccines named GP5m-Ft and (Bp-IVp)_3_-Ft, respectively. Through a comparison with an inactivated vaccine in mice, we found that the GP5m-Ft vaccine elicited effective humoral and cellular immune responses against PRRSV. Additionally, pigs vaccinated with the GP5m-Ft vaccine exhibited a shorter duration of illness, milder symptoms, and slower transmission compared to unvaccinated pigs. These findings suggest that GP5m-Ft is a promising vaccine candidate for controlling PRRS.

## 2. Materials and Methods

### 2.1. Animals and Ethics Statement

The experiment utilized 5–6-week-old SPF-grade female BALB/c mice, which were procured from the Animal Experiment Center of Huazhong Agricultural University and bred at the Experimental Center of Huazhong Agricultural University. The 4-week-old piglets were obtained from a pig farm in Hubei Province, and the sows underwent testing using RT-qPCR and ELISA, with both the PRRSV antigen and antibody yielding negative results. All animal experiments conducted in this study received approval from the Animal Ethics Committee of the Huazhong Agricultural University Laboratory, with the approval ID number HZAUMO-2023-0305 for mice and HZAUSW-2023-0068 for pigs.

### 2.2. Virus, Cells, and Reagents

The PRRSV strain WUH3 is a highly pathogenic PRRSV (GenBank accession number HM853673) [35,36]. African green monkey kidney cells (MARC-145) were purchased from the China Center for Type Culture Collection (Wuhan, China) and cultured in Dulbecco’s Modified Eagle Medium (DMEM) supplemented with 10% fetal bovine serum (FBS) in a 5% CO₂ incubator. *Escherichia coli* DH5α, and BL21 (DE3) cells were obtained from the Beijing Biomed Co., Ltd. (Beijing, China). The HRP-conjugated goat anti-mouse IgG antibody and anti-6 × His-tag monoclonal antibody were purchased from the Shanghai Beyotime Biotechnology Co., Ltd. (Shanghai, China). The necessary reagents, including DNA markers, protein markers, and enhanced chemiluminescence (ECL) solutions, were bought from the Beijing Solarbio Biotechnology Co., Ltd. (Beijing, China). 

### 2.3. Expression and Purification of Recombinant Proteins

In order to enhance the immunogenicity of the PRRSV GP5 subunit vaccine, we replaced the decoy epitope (aa 27–31) of GP5 in the highly pathogenic PRRSV strain WUH3 with neutralizing epitope B (aa 37–45). In addition, mutations were introduced at four glycosylation sites (N30, N34, N35, and N51) to generate the modified GP5 (GP5m). Furthermore, a fusion gene, Bp-IVp, was created by linking the GP5 B epitope, a pan-allelic malaria universal T-cell epitope (PADRE), and the GP5 IV epitope (aa 186–200). The GP5m gene and the three repeated Bp-IVp genes were fused to the ferritin gene derived from *H. pylori* ferritin (GenBank accession no. NP_223316) [37], respectively. This resulted in the construction of two prokaryotic expression plasmids: pET-15b-GP5m-Ft and pET-15b-(Bp-IVp)_3_-Ft. Another plasmid, pET-15b-GP5m was constructed for the sole expression of GP5m. The DNA sequences encoding GP5m, (Bp-IVp)_3_, and ferritin were synthesized and inserted into the prokaryotic expression vector pET-15b (GenScript, Nanjing, China). *Escherichia coli* BL21 (DE3) cells were transformed with the recombinant plasmids pET-15b-GP5m, pET-15b-GP5m-Ft and pET-15b-(Bp-IVp)_3_-Ft, respectively. GP5m-Ft, (Bp-IVp)_3_-Ft, and GP5m expression were induced by incubating *E. coli* cells with 0.5 mM isopropyl-β-d-1-thiogalactopyranoside overnight at 37 °C. The bacterial precipitate was collected by centrifugation at 8000× *g* for 20 min. High-pressure homogenization was performed using a crusher (ATS engineering Ltd., Brampton, ON, Canada). Subsequently, the supernatant was collected following centrifugation at 15,000× *g* for 1 h. The protein was purified by a Ni NTA Beads 6FF affinity chromatography medium (Smart-Lifesciences, Changzhou, China) in 20 mM Tris-HCl (pH 9.0) and 150 mM sodium chloride. The target protein was eluted using 20 mM Tris-HCl (pH 9.0), 100mM Imidazole, and 150 mM sodium chloride. The eluted protein was concentrated to 10 mg/mL through ultrafiltration and then subjected to a Superose™ 6 Increase 10/300 GL column (GE Healthcare, Chicago, IL, USA) for molecular sieve chromatography, using phosphate-buffered saline (PBS) as the buffer at a flow rate of 0.5 mL/min. The final protein concentration was determined using a bicinchoninic acid (BCA) test kit (Beyotime, Shanghai, China). The purified recombinant proteins were stored at −80 °C until further use.

### 2.4. Detection of Purified Protein Using Western Blotting

The recombinant protein was separated using 12% SDS-PAGE and then was transferred to polyvinylidene difluoride membrane. The membrane was blocked with 5% skim milk in 0.05% tris-buffered saline containing Tween 20 (TBST) and was incubated with an anti-6 × His-tag monoclonal antibody (1:5000) and an HRP-conjugated goat anti-mouse IgG antibody (1:5000). Following three washes with TBST, protein bands were detected using an ECL system, and were analyzed using the ImageLab 4.0.1 software.

### 2.5. Dynamic Light Scattering (DLS)

We conducted dynamic light scattering (DLS) measurements using a Zetasizer Nano ZS instrument from Malvern Panalytical’s Zetasizer Nano series. The instrument was equipped with a 633 nm laser, and the scattering angle was set to 90°. We obtained appropriate amounts of GP5m-Ft, (Bp-IVp)_3_-Ft, and Ft proteins from purified samples, which were then diluted to a concentration of 10 μg/mL in PBS. Thorough mixing was ensured to obtain a homogeneous sample. Prior to DLS measurements, we typically performed a centrifugation step to remove any large particles or impurities suspended in the solution. The samples were centrifuged at 16,000× *g* for 10 min to precipitate the particles. Additionally, we employed 0.45-μm pore size filters to remove large particles before conducting DLS measurements. The DLS experiments were conducted at a temperature of 37 °C. Each protein sample was measured 3 times with 13 runs per measurement to ensure statistical significance. 

### 2.6. Transmission Electron Microscope (TEM)

For TEM negative staining observation, 10 µL of the GP5m-Ft, (Bp-IVp)_3_-Ft, and Ft proteins were dropped onto a carbon-coated copper grid and incubated for 5 min, respectively. Followed by adding 5 µL of uranyl acetate staining for 3–5 min. After air-drying, the samples were observed under a TEM (JEM-1200EX, JEOL, Tokyo, Japan).

### 2.7. Vaccination in Mice

The purified protein (GP5m-Ft, (Bp-IVp)_3_-Ft, or GP5m) or PBS was mixed in a ratio of 6:1 (mass ratio) with aluminum hydroxide adjuvant (Solarbio, Beijing, China), respectively, to form an emulsion. The PRRSV (WUH3 strain) inactivated vaccine was provided by Wuhan Keqian Biology Co., Ltd. (Wuhan, China). 5-6-week-old female BALB/c mice were divided into five groups (ten mice in each group) and were administered with these vaccines (20 μg per mouse) on day 0 and day 21 post-primary immunization. Blood samples were collected from the mice on day 21 and day 42 to detect GP5m-specific IgG antibodies and serum-neutralizing antibody titers. On day 42, three mice from each group were sacrificed to isolate spleen tissues and serum samples. All mice were euthanized according to the appropriate animal welfare protocol.

We used the same batch of vaccines for both primary vaccination and booster vaccination in the mice and pigs. The vaccine underwent stability evaluations, including pH stability, temperature stability, particle aggregation, and sedimentation, as well as long-term storage stability. The results demonstrated that the vaccine exhibited good stability within a certain time period, and using the same batch did not have adverse effects in both species.

### 2.8. Vaccination and PRRSV Challenge in Piglets

Prior to vaccination, ten 4–week-old piglets were detected by ELISA and confirmed to be seronegative for PRRSV antibody. Furtherly, they were determined to be negative for PRRSV, Porcine parvovirus, Porcine circovirus, and swine influenza virus by RT-qPCR. Piglets were randomly divided into two groups (each group consisting of five piglets). The experimental group received intramuscular injections of a mixture of purified GP5m-Ft protein and aluminum hydroxide adjuvant on days 0, 14, and 28 (200 μg per piglet), and the control group received intramuscular injections of a mixture of PBS and aluminum hydroxide adjuvant. Blood samples were collected from each group on days 14, 28, and 38 to examine the serum-neutralizing antibody titers against PRRSV. On day 38, all piglets were intramuscularly injected with a dose of 2 × 10^5^ TCID_50_ of a high pathogenic PRRSV strain WUH3. The rectal temperatures of all piglets were recorded daily, and the survival rate was measured on day 14 after PRRSV challenge. Serum samples were collected at 1, 3, 5, 7, 10, and 14 days post-challenge (dpc) to detect serum viral load. All piglets were euthanized on day 14 dpc, and lung tissues were collected for histopathological examination.

### 2.9. Enzyme-Linked Immunosorbent Assay (ELISA)

The purified GP5m protein was diluted with 50 mM carbonate buffer (pH 9.6) to the optimal coating concentration (1 μg/mL) and was then added to the enzyme plate at 4 °C overnight. After washing with PBST, blocking solution was added at 37 °C for 1 h. The serum for ELISA detection was first diluted with PBS to 1:64 and then subjected to serial two-fold dilutions, followed by incubation at 37 °C for 1 h. After another wash with PBST, HRP-conjugated goat anti-mouse IgG (1:5000) was added for 1 h at 37 °C. Following the washing step, TMB was added and incubated for 15 min in the absence of light. Then, stop solution was added to the enzyme plate wells to terminate the reaction, after which the OD value was recorded on a microplate reader at a wavelength of 630 nm.

### 2.10. PRRSV Serum Neutralization Assays

Prior to testing, the serum samples from the mice and piglets were inactivated using a 56 °C water bath for 30 min and then diluted with PBS by serial two-fold. The diluted sera were mixed with an equal volume of the PRRSV strain WUH3 (200 TCID_50_) for 1 h at 37 °C, and the mixture was added to MARC-145 cells for an additional 1 h. Subsequently, the supernatants were discarded, and fresh DMEM medium containing 2% FBS was added to the plate, which was then kept at 37 °C with 5% CO₂. The cytopathic effect (CPE) was observed for 5 days. The titer of the neutralizing antibody against PRRSV was calculated according to the Reed-Muench’s method.

### 2.11. Spleen Lymphocyte Proliferation Test in Mice

A total of three mice from each group were sacrificed to obtain splenic lymphocytes. These fresh lymphocytes were cultured in 96-well plates with RPMI-1640 medium containing 10% FBS at 37 °C with 5% CO_2_. Furthermore, we added concanavalin A (Con A) or GP5m protein at a concentration of 5 µg/mL into the spleen lymphocytes to stimulate for another 44 h. Subsequently, 10 μL of Cell Counting Kit-8 (CCK-8) solution was added to each well for 4 h. The plates were read at a wavelength of 450 nm using a microplate reader (BioTek, Winooski, VT, USA). The stimulation index (SI) was calculated according to the following formula: the ratio of the optical density value of the well containing stimulated cells to the OD value of the well containing unstimulated cells.

### 2.12. ELISpot Analysis of Interferon-Gamma (IFN-γ) in Mice Splenic Lymphocytes 

In order to assess specific T lymphocyte responses in the mice immunized with vaccines, ELISpot analysis was conducted. A total of 200 μL of RPMI-1640 medium was dispensed into precoated plates utilizing an IFN-γ/ELISpot assay kit (Dakewe, Shenzhen, China). The plates were then incubated at room temperature for 5 to 10 min to facilitate activation. In order to ensure the vitality of splenocytes, a viability dye exclusion method was employed, confirming that over 98% of the cells in all samples were viable. Next, a 100 μL suspension of splenic lymphocytes (1 × 10^6^ cells) was added to each well, and an equal volume of RPMI-1640 medium served as the background negative control. The protein concentration was standardized to 0.8 μg/mL using culture medium. Subsequently, 10 μL of recombinant GP5m protein was introduced to each detection well, 10 μL of a positive stimulus solution was added to the positive control wells, and 10 μL of RPMI-1640 medium was dispensed into both the negative and background control wells. The cells were then incubated for 18 h at 37 °C in a 5% CO₂ incubator. Following this incubation period, the analysis proceeded in accordance with the manufacturer’s instructions.

### 2.13. Testing PBMC Proliferation in Piglets

In each group, five piglets were aseptically sampled to collect jugular venous blood. Lymphocyte separation solution (Dakewe, Shenzhen, China) was used to isolate peripheral blood mononuclear cells (PBMCs). These fresh lymphocytes were then cultured in 96-well plates with RPMI-1640 medium supplemented with 10% Fetal Bovine Serum (FBS) at 37 °C with 5% CO_2_. Additionally, PBMC lymphocytes were stimulated with concanavalin A (Con A) or GP5m at a concentration of 5 µg/mL for 44 h. Following this, Cell Counting Kit-8 (CCK-8) solution was added to each well and was incubated for 4 h. The plates were then read at a wavelength of 450 nm using a microplate reader (BioTek, Winooski, VT, USA). The stimulation index (SI) was calculated using the following formula: the ratio of the optical density value of the well containing stimulated cells to the OD value of the well containing unstimulated cells.

### 2.14. Clinical Observation

After the pigs were infected with PRRSV, their physical condition was regularly monitored, and the clinical severity of respiratory disease was scored daily from 0 (normal) to 6 (severe respiratory distress and abdominal breathing) [21]. Rectal temperatures were measured by the same person at the same time each day.

### 2.15. Quantitative Analysis of Viral Loads

After collecting serum samples, we employed real-time quantitative PCR (qPCR) technology to precisely quantify the viral load. Initially, the total RNA in the serum was reverse-transcribed into cDNA using RT-PCR. The copy numbers of the standards were serial 10-fold dilutions. The reaction conditions were 50 °C for 2 min, followed by an initial denaturation at 95 °C for 10 min before entering the cycling steps. The cycling conditions were 95 °C for 15 s and 60 °C for 1 min for a total of 45 cycles. The quantitative fluorescence reaction system is as follows. Premix Ex Taq (2×): 1.5 µL. Upstream primer and downstream primer (10 µM): 0.5 µL each. TaqMan probe (3 µM): 1 µL (the specific sequences are listed in the Table 1). cDNA: 2.0 µL. And finally, RNase Free H_2_O was added to a suitable volume to reach a final volume of 25.0 µL.

### 2.16. Histological Observation

The lung tissues were fixed in 4% paraformaldehyde for 24 h, followed by embedding in paraffin for sectioning. The histological alterations observed in the sections were documented using hematoxylin–eosin (HE) staining under an optical microscope (Nikon, Tokyo, Japan).

### 2.17. Statistical Analysis

The data were analyzed using GraphPad Prism v8.01 (GraphPad Software, La Jolla, CA, USA). For those instances where two groups were being compared, the data were analyzed using the Student’s *t*-test. In contrast, when more than two groups were being compared, the data were analyzed using a one-way analysis of variance (ANOVA). The data are presented as mean ± SEM in each group. ns indicates *p* ≥ 0.05, * indicates *p* < 0.05, ** indicates *p* < 0.01, and *** indicates *p* < 0.001.

## 3. Results

### 3.1. Design, Purification, and Assembly Validation of Recombinant Nanoparticles

**Figure 1:**
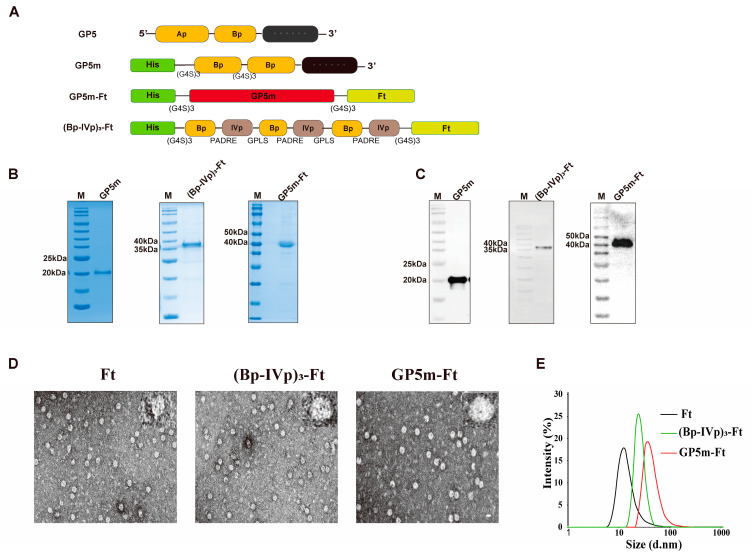
Design, purification, and assembly validation of recombinant proteins. (**A**) Schematic diagrams of GP5, GP5m, GP5m-Ft and (Bp-IVp)_3_-Ft. Ap represents the decoy epitope of GP5 (aa 27–31); Bp represents the neutralizing epitope B (aa 37–45); IVp represents the IV epitope of GP5 (aa 186–200). (**B**) SDS-PAGE analysis of purified GP5m, GP5m-Ft and (Bp-IVp)_3_-Ft proteins. GP5m, (Bp-IVp)_3_-Ft and GP5m-Ft consist of 182 aa, 293 aa and 363 aa, respectively. (**C**) Western blot analysis of purified GP5m-Ft and (Bp-IVp)_3_-Ft proteins using 6 × His-tag antibody. (**D**) TEM images of recombinant Ft, GP5m-Ft, and (Bp-IVp)_3_-Ft proteins. (**E**) DLS analysis of Ft, GP5m-Ft, and (Bp-IVp)_3_-Ft proteins.

We fused the GP5m gene and the three tandem Bp-IVp genes to the Ft gene to construct the prokaryotic expression plasmids pET-15b-GP5m-Ft and pET-15b-(Bp-IVp)_3_-Ft. The structure of the target antigens is depicted schematically in Figure 1A. The purified GP5m, GP5m-Ft, and (Bp-IVp)_3_-Ft were detected by using SDS-PAGE with Coomassie bright blue staining (Figure 1B). Western blot analysis demonstrated that these proteins could be detected using anti-6 × His-tag monoclonal antibody (Figure 1C). Through performing TEM and DLS, we examined whether GP5m-Ft and (Bp-IVp)_3_-Ft could self-assemble into nanoparticles. As shown in Figure 1D, we observed many spherical nanoparticles of relatively uniform size in both GP5m-Ft and (Bp-IVp)_3_-Ft proteins. Consistently, the DLS results revealed that the average diameter of Ft nanoparticles was 12.6 nm, and the average diameters of the (Bp-IVp)_3_-Ft and GP5m-Ft nanoparticles were 22.9 nm and 26.3 nm, respectively (Figure 1E). Together, these data indicate that recombinant (Bp-IVp)_3_-Ft and GP5m-Ft proteins can self-assemble into nanoparticles, and that the size of the nanoparticles is positively correlated with the size of the displayed target antigen.

### 3.2. Humoral Immune Responses of (Bp-IVp)_3_-Ft and GP5m-Ft in Mice

**Figure 2:**
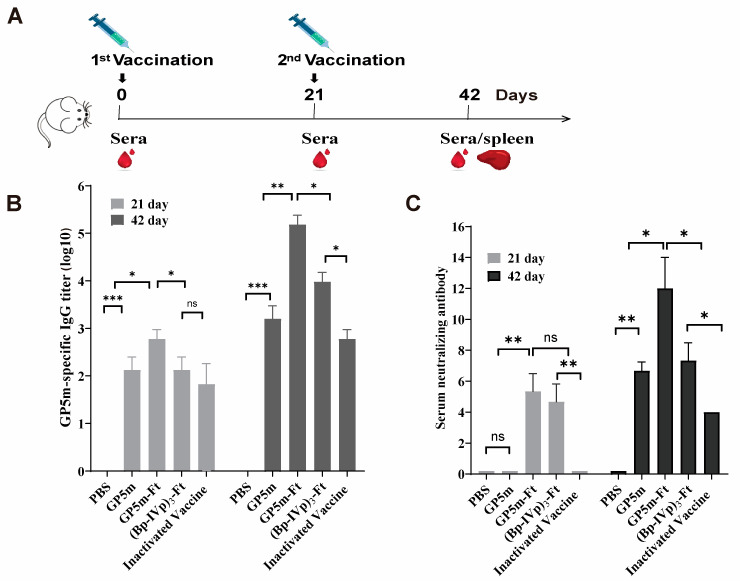
Humoral immune responses of recombinant protein vaccines in mice. (**A**) Schematic diagram of the immunization procedures for mice. (**B**) Detection of GP5m-specific ELISA antibody of the immunized mice at 21 and 42 dpi. (**C**) Serum neutralizing antibody levels of the vaccine-immunized mice at 21 and 42 dpi. The serum-neutralizing antibody titers were determined using an end-point dilution reduction assay. The data are presented as mean ± SEM in each group. ns indicates *p* ≥ 0.05, * indicates *p* < 0.05, ** indicates *p* < 0.01, and *** indicates *p* < 0.001.

We further evaluated the immunogenicity of the (Bp-IVp)_3_-Ft and GP5m-Ft vaccines in BALB/c mice. As controls, we included the inactivated PRRSV vaccine and the recombinant GP5m protein. The immunization procedure is illustrated in Figure 2A. Blood samples were collected on days 21 and 42 after the initial immunization to measure serum-specific IgG antibodies against GP5m and serum neutralizing antibodies against PRRSV. As depicted in Figure 2B, GP5m-specific IgG antibodies were detectable in all vaccinated groups at 21 dpi, with significantly increased antibody levels observed in all groups at 42 dpi following booster administration. Notably, GP5m-Ft elicited the highest levels of GP5m-specific antibodies among the four tested vaccines. Similarly, the serum neutralization results demonstrated that GP5m-Ft induced the highest serum neutralizing antibody titer, reaching 1:10.2, followed by (Bp-IVp)3-Ft with an average neutralizing antibody titer of 1:7.2 (Figure 2C). In both antibody assays, the levels induced with the inactivated PRRSV vaccine and recombinant GP5m vaccine were significantly lower than those elicited by the two nanoparticle vaccines. Overall, these findings indicate that ferritin fusion can markedly enhance PRRSV GP5 immunogenicity.

### 3.3. Cellular Immune Response of (Bp-IVp)_3_-Ft and GP5m-Ft in Mice

**Figure 3:**
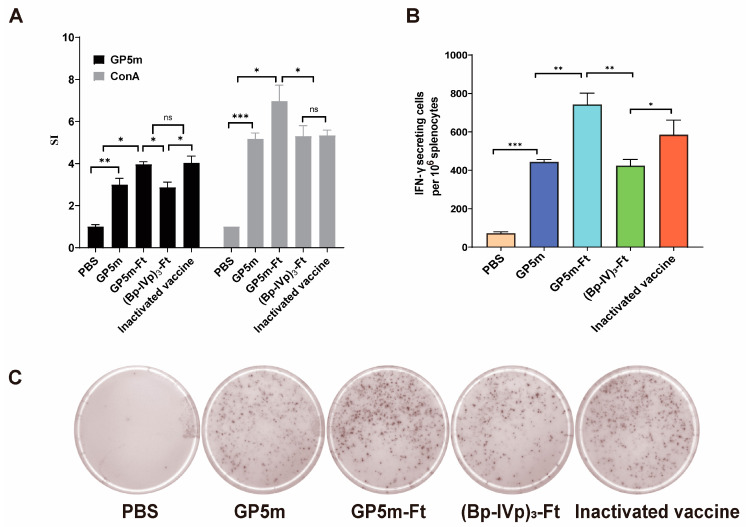
Cellular immune responses of vaccinated mice. (**A**) Lymphocyte proliferation in response to different immunogens. The isolated mice splenocytes were stimulated in vitro with Con A or GP5m. The stimulation index was measured using the CCK8 assay. (**B**,**C**) Levels of IFN-γ secreted by mouse splenic lymphocytes detected using ELISpot. (**B**) Number of lymphocyte clones secreting IFN-γ. (**C**) Spotted holes of lymphocytes secreting IFN-γ. The data are presented as mean ± SEM in each group. ns indicates *p* ≥ 0.05, * indicates *p* < 0.05, ** indicates *p* < 0.01, and *** indicates *p* < 0.001.

In addition to detecting the antibody levels, we further evaluated the cellular immune activation of the vaccine by assessing the lymphocyte efficiency and IFN-γ production of the immunized mice. When compared with the PBS group, the lymphocyte levels of all recombinant proteins or inactivated vaccine groups significantly increased (Figure 3A). The SI values of the GP5m-Ft group were the highest among the four tested vaccines. By performing ELISpot, we found that the number of IFN-γ secreting cells in GP5m-Ft group was significantly higher than that of other vaccine-immunized groups (Figure 3B,C). Together, these findings further indicate that the fusion of ferritin can significantly enhance the cellular immune response of PRRSV GP5.

### 3.4. Evaluation of the Immunogenicity of GP5m-Ft Vaccine in Piglets

**Figure 4:**
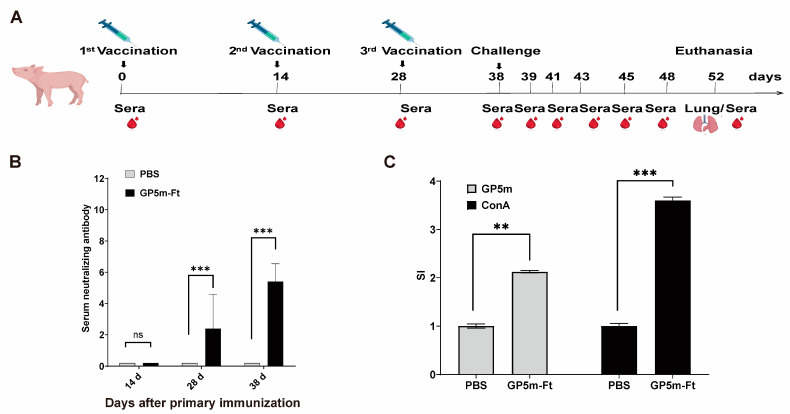
Immunogenicity evaluation of GP5m-Ft in piglets. (**A**) Schematic diagram of the immunization/challenge procedures for the piglet experiments. (**B**) Sera were collected at 14, 28, and 38 dpi to determine the titers of neutralizing antibodies against PRRSV. The titers of neutralizing antibodies were determined using an end-point dilution reduction assay. (**C**) Lymphocyte proliferation response of peripheral blood mononuclear cells isolated from the immunized piglets at 38 dpi. The isolated peripheral blood mononuclear cells were stimulated in vitro with GP5m or ConA, and the stimulation index was measured using the CCK8 assay. The data are presented as mean ± SEM in each group. ns indicates *p* ≥ 0.05, ** indicates *p* < 0.01, and *** indicates *p* < 0.001.

From the experimental results of the mice, it can be seen that GP5m-Ft protein has the strongest immunogenicity. Thus, we chose GP5m-Ft to evaluate its ability to protect piglets against PRRSV infection. The vaccination and PRRSV challenge process on piglets is depicted in Figure 4A. As shown in Figure 4B, the average serum neutralizing antibody titers reached 1:5.4 at 38 dpi. In addition, when compared to the PBS control group, the SI values of lymphocytes in the GP5m-Ft-immunized group were significantly higher irrespective of the stimulation with GP5m protein or Con A (Figure 4C). These findings suggest that GP5m-Ft can also stimulate humoral and cellular immunity in piglets.

### 3.5. Immunization with GP5m-Ft Vaccine Provides Effective Protection against PRRSV Challenge in Piglets

**Figure 5:**
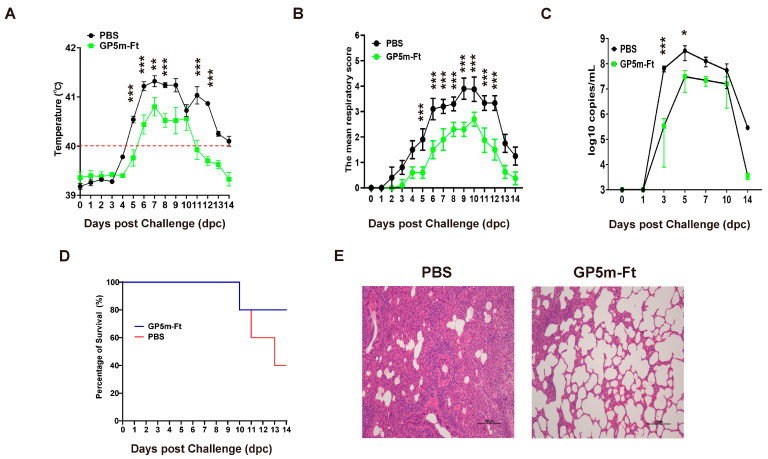
Protection efficacy of GP5m-Ft vaccine against PRRSV challenge in piglets. (**A**) The mean rectal temperatures of immunized piglets at 0–14 dpc. (**B**) The mean respiratory scores at 0–14 dpc. (**C**) The viral loads in the serum samples collected at 0, 1, 3, 5, 7, 10, and 14 dpc were determined using qPCR. (**D**) Survival curves of the piglets within 14 days after PRRSV challenge. (**E**) Histopathological examination of lung sections following PRRSV challenge experiments in piglets (hematoxylin and eosin-stain; bar = 200 μm). The data are presented as mean ± SEM in each group. * indicates *p* < 0.05, ** indicates *p* < 0.01, and *** indicates *p* < 0.001.

In order to investigate whether immunization using the GP5m-Ft vaccine can provide protection against PRRSV challenge, all piglets were intramuscularly injected with 2 × 10^5^ TCID_50_ of the highly pathogenic PRRSV strain WUH3 at 38 days after the first immunization. For piglet body temperature, during 0–4 dpc and 9–10 dpc, there were no significant differences between the GP5m-Ft-immunized piglets and the PBS-immunized piglets. However, the body temperature of the GP5m-Ft-immunized piglets was significantly lower in comparison to that of the PBS-immunized piglets during 5–8 dpc and 11–12 dpc. For mean respiratory score, significant differences between the GP5m-Ft-immunized piglets and the PBS-immunized piglets were observed at 5–12 dpc, with no obvious differences detected at 1–4 dpc (Figure 5B). 

In addition, the serum viral load of the GP5m-Ft-immunized piglets was significantly lower than that of the PBS-immunized piglets during 3–5 dpc. At 0, 1, 7, 10, and 14 dpc, there were no observed serum viral loads between the GP5m-Ft group and the PBS group (Figure 5C). Similarly, the survival rate of GP5m-Ft-immunized piglets at 14 dpc was 80%, whereas the survival rate of the PBS-immunized group was only 40% (Figure 5D). The results of the lung pathology sections showed that the PBS group had marked congestion and severe interstitial proliferative pneumonia, which are typical signs of PRRSV infection. In contrast, piglets immunized with GP5m-Ft showed limited infection in the lungs (Figure 5E). In conclusion, these data suggest that vaccination with the GP5m-Ft is effective at protecting piglets against PRRSV infection.

## 4. Discussion

PRRSV is a highly mutagenic RNA virus. Therefore, the updating of vaccine strains is of significant importance for the prevention of PRRSV [38,39]. The current commercial PRRSV vaccines have numerous limitations, including the need for high-dose vaccination, high cost, limited protection against homologous strains, and weak or no cross-protection against heterologous strains [8,10]. The GP5 protein is a major structural component of PRRSV, and possesses notable immunogenicity. It has the capacity to induce the production of neutralizing antibodies [17,40,41,42]. However, vaccines based on the original GP5 protein generally suffer from the defect of being unable to induce high levels of neutralizing antibodies [43,44,45]. In this study, we designed a novel GP5 vaccine for PRRSV called GP5m-Ft. This vaccine can effectively stimulate humoral and cellular immunity in mice and pigs.

Due to the low immunogenicity of low-molecular-weight proteins, it is often necessary to use suitable protein carriers to present neutralizing epitopes. Over the years, studies have demonstrated that nanoparticles offer significant advantages in the design and development of vaccines [46,47,48,49]. Several studies have been conducted with the aim of enhancing the innate immune response by utilizing antigen-delivery platforms that mimic pathogen properties and subsequently trigger adaptive immunity [50,51,52]. Ferritin, a widely distributed protein in organisms, plays a crucial role in iron metabolism, and is considered to be an excellent protein nanocage due to its exceptional stability and biocompatibility [29,53].

As stated above, (Bp-IV)_3_-Ft contains PADRE T cell epitopes, which may contribute to higher T cell immunity. Compared to GP5m, the GP5m-Ft vaccine induced a higher cellular immune response, indicating that ferritin nanoparticles promote the immune responses of GP5m not only for cellular immune responses, but also for humoral immune responses. In addition, in previous studies, while protein-based nanoparticle vaccines, such as ferritin nanoparticles, may have lower chances of inducing T cell immunity compared to viral vector or nucleic acid-based vaccines, they can still elicit T cell responses under certain circumstances. Some studies have shown that protein-based nanoparticles, including ferritin nanoparticle, have the ability to undergo cross-presentation [21,34]. 

The groundbreaking research conducted by Ma [21] revealed that the GP5m-ferritin vaccine induced a significantly superior immune response in pigs compared to the traditional inactivated PRRSV vaccine. Not only did the vaccine trigger higher serum antibody titers, but it also significantly boosted Th1-type cellular immune responses. Importantly, pigs vaccinated with GP5m-ferritin exhibited significantly lower body temperatures, respiratory scores, viremia, and lung lesion scores than the unvaccinated controls when challenged with the virus. These compelling results by Ma [21] strongly demonstrate that the GP5m-Ft subunit vaccine is capable of eliciting specific protective immune responses and is, therefore, considered to be a highly potential vaccine candidate.

Inspired by this study, we conducted immunization and challenge protection tests in piglets using the purified GP5m-Ft vaccine. Compared with the baculovirus expression system, the vaccine elicited a similar cellular immune response in pigs, although the antibody titers were lower. The ELISpot results showed a significantly enhanced immune response to specific T lymphocytes. Furthermore, the temperature, viremia, and lung lesion scores of the vaccinated pigs were consistent with those observed by Ma [21]. These findings not only indicate that the prokaryotic-expressed GP5m-Ft nanoparticle vaccine has a significant advantage in reducing adjuvant dependence, but also demonstrate its efficacy in challenge protection. 

In our research, we were astonished to discover that the immune response of pigs is weaker compared to that in mice. This difference in immune response is primarily attributed to several core factors, with the most critical factor being the inherent nature of the immune systems in different species. Pigs and mice exhibit variations in immune characteristics, and their responses to vaccines may differ due to variances in the diversity of immune cell populations, antigen presentation methods, and immune signal transduction pathways. Additionally, the vaccine formulation, delivery method, and use of adjuvants can also exert a significant impact on the observed immune response, leading to differences in immune responses between different species [54,55,56]. In fact, disparities in both the degree and nature of the immune response induced by vaccines across various species are not uncommon. In order to ensure experimental accuracy, we utilized identical vaccine formulations for both mice and pigs, while striving to minimize any potential differences during vaccine preparation processes, thus ensuring the reliability of the experimental results. However, further research efforts are still required for a more comprehensive understanding of dynamic changes in immune responses and the optimization of vaccine strategies for different animal species. This will aid us in gaining a more precise grasp of the complex mechanisms underlying immune responses and provide a solid scientific foundation for future vaccine development.

## 5. Conclusions

This study presented a novel design for a PRRSV subunit vaccine. The recombinant GP5m-Ft protein can be expressed in *E. coli* and is easily purified. In vitro, the purified GP5m-Ft can self-assemble into nanoparticles with a diameter of about 26.3 nm. Immunization with GP5m-Ft in mice induced higher levels of neutralizing antibodies compared to the inactivated PRRSV vaccine, and this also promoted the proliferation and activation of mouse lymphocytes. The results of challenge studies in piglets indicated that GP5-Ft could effectively resist infection from highly pathogenic PRRSV and significantly improve the survival rate of piglets. These findings demonstrate that GP5m-Ft has potential as a candidate vaccine against PRRSV infection.

## Figures and Tables

**Table 1:** The qPCR primers and probe of PRRSV.

Primer and Probe	Sequence (5′-3′)
PRRSV N Forward	TTCCCTCTAGCGACTGAAGA
PRRSV N Reverse	AGTTCCAGCACCCTGATTG
Probe 6 FAM	GCAATTGTGTCTGTCGTCGATCCAGA-BHQ1

## Data Availability

The datasets generated in this study are available upon request.
